# Preference-Based Implementation of Video Consultations in Urban and Rural Regions in Outpatient Care in Germany: Protocol for a Mixed Methods Study

**DOI:** 10.2196/50932

**Published:** 2024-04-11

**Authors:** Lara Kleinschmidt, Anke Walendzik, Jürgen Wasem, Klemens Höfer, Beatrice Nauendorf, Matthias Brittner, Paul Brandenburg, André Aeustergerling, Udo Schneider, Anja Wadeck, Stephanie Sehlen, Sebastian Liersch, Katharina Schwarze, Carsten Schwenke, Theresa Hüer

**Affiliations:** 1 Institute for Health Care Management and Research University of Duisburg-Essen Essen Germany; 2 Kassenärztliche Vereinigung Berlin Berlin Germany; 3 Kassenärztliche Vereinigung Westfalen-Lippe Dortmund Germany; 4 Kassenärztliche Vereinigung Schleswig-Holstein Bad Segeberg Germany; 5 Kassenärztliche Vereinigung Mecklenburg-Vorpommern Schwerin Germany; 6 Techniker Krankenkasse Hamburg Germany; 7 AOK Nordost Potsdam Germany; 8 AOK NordWest Dortmund Germany; 9 SCO:SSiS Minden Germany

**Keywords:** study protocol, video consultation, preference elicitation, discrete choice experiment, implementation, telemedicine, teleconsultation, e-consultation, outpatient, rural area, remote, preferences, strategy

## Abstract

**Background:**

Particularly in rural regions, factors such as lower physician density and long travel distances complicate adequate outpatient care. However, urban regions can also be affected by deficits in care, for example, long waiting times. One model of care intending to improve the situation is the implementation of video consultations. The study protocol presents the methodology of the research project titled “Preference-based implementation of the video consultation in urban and rural regions” funded by the German Federal Joint Committee (funding number 01VSF20011).

**Objective:**

This study aims to identify existing barriers to the use of video consultation and the preferences of insured individuals and physicians as well as psychotherapists in order to optimize its design and thus increase acceptance and use of video consultations in urban and rural regions.

**Methods:**

Built on a mixed methods approach, this study first assesses the status quo of video consultation use through claims data analysis and carries out a systematic literature review on barriers and promoting factors for the use of video consultations. Based on this preliminary work, focus groups are conducted in order to prepare surveys with insureds as well as physicians and psychotherapists in the second study phase. The central element of the survey is the implementation of discrete choice experiments to elicit relevant preferences of (potential) user groups and service providers. The summarized findings are discussed in a stakeholder workshop and translated into health policy recommendations.

**Results:**

The methodological approach used in this study is the focus of this paper. The study is still ongoing and will continue until March 2024. The first study phase has already been completed, in which preliminary work has been done on potential applications and hurdles for the use of video consultations. Currently, the survey is being conducted and analyses are being prepared.

**Conclusions:**

This study is intended to develop a targeted strategy for health policy makers based on actual preferences and perceived obstacles to the use of video consultations. The results of this study will contribute to further user-oriented development of the implementation of video consultations in German statutory health insurance. Furthermore, the iterative and mixed methods approach used in this study protocol is also suitable for a variety of other research projects.

**International Registered Report Identifier (IRRID):**

DERR1-10.2196/50932

## Introduction

Particularly in rural regions, factors such as lower physician density and long travel distances complicate adequate outpatient care [1]. However, urban regions can also be affected by deficits in care, for example, due to long waiting times [2]. One model of care that could improve the situation is the implementation of video consultations in outpatient care, meaning online contact in real time with video transmission between physician or psychotherapist and patient. In addition to questioning about symptoms and medical history (anamnesis), signs of illness can be examined, and—possibly—a diagnosis can be made. Particularly in the case of long journeys, for follow-up appointments (for example after minor surgeries), or for the provision of repeated prescriptions, video consultations can be a useful tool and the insured individual does not have to visit the doctor’s office for every appointment. In the field of psychotherapy, therapeutic or certain diagnostic sessions can also take place in the form of video consultations [3].

Several studies provide preliminary clinical evidence of the benefits of telemedicine in certain patient populations, such as the chronically ill or patients living in nursing homes. A project in a predominantly rural region in Germany demonstrated the effectiveness of video consultations for nursing home residents. In particular, routine appointments, such as follow-up treatments, could be carried out efficiently remotely. Advantages for all parties involved were demonstrated through improved access to general practitioners (GPs) and specialists, a reduction in hospital admissions, and the elimination of stressful journeys and time-consuming home visits [4]. For people with mental disorders such as depression, telemedicine has proven to be a promising tool to enhance quality of life and access to treatment [5-7]. In addition, high patient satisfaction rates with video consultations could be shown in the field of orthopedics [8,9].

Since the health care system in Germany is predominately based on a social insurance system, in which 88.3% of the German population is insured [10], the project focuses on the statutory health insurance (SHI) system. In the German SHI, since April 2017, video consultations could initially only be provided by a few specialist groups and only for selected indications and follow-up appointments. In the second quarter of 2019, these restrictions were mostly abolished and the assessment of the appropriateness of a diagnosis or treatment via video consultation was placed in the decision-making responsibility of the physician [3,11]. During the COVID-19 pandemic, there were numerous (primarily temporary) adjustments in the regulations for their provision. Video consultations are part of the SHI benefits catalog. The remuneration of video consultations in outpatient medical care in the German SHI system is not fee-for-service based, but predominantly part of age-differentiated quarterly flat rates, which cover the entire medical care of a patient (not just video consultations) by the attending physician during this period. In addition, there are supplementary payments that compensate for the additional technical effort and the digital authentication of an unknown patient in video consultations [3].

However, since 2017 and up to the beginning of the COVID-19 pandemic, video consultations have hardly played a role in outpatient medical care in Germany [11]. Many physicians feared an estrangement of the physician-patient relationship and that as a consequence this would result in the physician's inability to meet the patients' needs. In addition, some assume that the reduced sensory perception on the screen and the lack of a holistic perspective could lead more easily to treatment errors or misdiagnoses. Concerns are also expressed about data protection and data security [12]. A study by Noweski et al [13] also showed that personal contact with the doctor is given high priority by the insured individual and that the insured individual only accepts digital doctor-patient consultations as an alternative in part or under specific conditions.

Thus, the central prerequisite for the creation of acceptance—related to the willingness to offer video consultations, but also to the willingness to make use of them—is the consideration of the preferences and perceived barriers of those involved. For this reason, this study addresses the question of how video consultations should be designed to improve situations of outpatient medical care while taking into account the preferences of the insured individual and physicians as well as psychotherapists. Weinhold and Gurtner [14] showed that patient satisfaction in primary care in rural regions depends on different factors than in urban regions. In order to be able to develop targeted strategies, a comparative preference assessment between rural and urban regions is carried out.

This study aims to address the following research questions:

- How often and for which treatment occasions are video consultations used in outpatient medical care in the SHI system in Germany? What are the characteristics of the user groups in rural and urban areas (use of video consultations in standard care)?
- What findings can be drawn from national and international studies on the implementation of video consultation with regard to best practices so far?
- Under what conditions (expectations of form and content, inhibiting and promoting factors) do insured individuals accept the digitalization of their medical consultations?
- Which application options do physicians and psychotherapists prefer?
- How do the preferences differ among GPs, psychotherapists, and other specialist groups?
- How do the preferences of insured individuals and the physicians as well as psychotherapists differ between urban and rural regions?
- Which kind of implementation of video consultations should be pursued in rural and urban regions?
- Where are the hurdles that need to be overcome?
- Is there a need for regulatory adaptations?

So far, there are no research results on the use of video consultations that consider users (patients) and providers (physicians/psychotherapists) on the one hand and compare preferences between urban and rural regions on the other hand. To elicit these preferences, this study uses the particularly suitable method of discrete choice experiments (DCE), which, in contrast to other instruments of preference elicitation, allow for an intuitive decision-making process by evaluating alternatives as a bundle of their characteristics. A DCE also offers the advantage of a realistic assessment situation, which avoids the problem of compositional methods, which is that respondents tend to rate all attributes as very important [15].

## Methods

### Study Design

**Figure 1:**
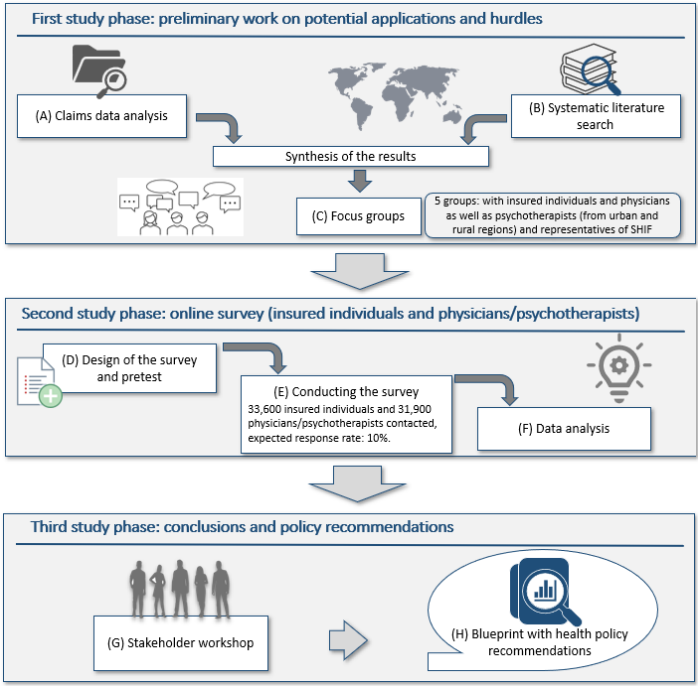
Study design and course of the study from April 2021 to March 2024. SHIF: statutory health insurance fund.

In order to develop a strategy for the preference-based implementation of video consultations in rural and urban regions, this study uses a mixed methods approach with a particular focus on DCE. The following Figure 1 shows the course of the study. The duration of the study is 3 years, beginning in April 2021 and continuing prospectively until March 2024.

In the first study phase, the status quo of the use of video consultations is examined by a claims data analysis (A). At the same time, a systematic literature review (B) on possible barriers and promoting factors on the use and provision of video consultation is conducted. Then, preliminary findings are discussed in focus groups (C) in order to prepare the following surveys with DCE. In the second phase of the study, surveys including DCE are designed (D), conducted (E), and analyzed (F) to elicit preferences for the design of video consultation of insured individuals on the one hand and physicians as well as psychotherapists on the other hand, comparing rural and urban regions. In the third study phase, all results are combined and discussed in a stakeholder workshop (G) and policy recommendations (H) are developed.

### Claims Data Analysis (A)

In work package A, claims data are analyzed focusing on patterns of the use of video consultations as well as the characteristics of their user groups in rural and urban regions (status quo).

To depict both, the perspective of insured and service providers, claims data from 3 statutory health insurance funds (SHIF) and 4 regional associations of statutory health insurance physicians (ASHIP) are used from the period April 2017 to the end of 2020.

Data for insured individuals and physicians, as well as psychotherapists in 4 German regions (“Westfalen-Lippe,” “Mecklenburg-Vorpommern,” “Schleswig-Holstein,” and “Berlin”), are analyzed. These regions are chosen because they include both rural and urban districts. The classification as a rural or urban district is carried out using the interactive web app “INKAR: Indicators and Maps of Spatial and Urban Development” offered by the Federal Institute for Research on Building, Urban Affairs, and Spatial Development. Among other things, the settlement structure of the district is considered as a combination of the population density and the settlement area share [16].

After a preliminary analysis of the data by the SHIF and ASHIP according to a consented evaluation concept, only aggregated data are sent to the University of Duisburg-Essen for further analysis. There, the data are combined and further evaluated according to the research question.

Aggregated data of approximately 6 million insured living in 1 of the 4 selected regions are analyzed. In addition to data on the use of video consultation, patient data on sociodemographic characteristics and diagnostic records are included. To depict the perspective of service providers, data from 31,900 physicians and psychotherapists are analyzed. Information on specialist groups (eg, GPs, psychotherapists, and other specialist groups) and sociodemographic characteristics of users compared with nonusers are collected. Furthermore, the influence of the place of residence or practice location on the use of video consultation is examined.

Descriptive analyses, differentiated according to subgroups (insured: age group, gender, employment situation, and type of region; physicians/psychotherapists: specialist group, age group, gender, type of operation and employment, and type of region) are conducted. Since the data are not provided at the individual level, the analysis of the aggregated data is performed using Excel (version 16.0; Microsoft Corp).

### Systematic Literature Review (B)

A systematic literature review including national and international studies on the application settings of video consultation as well as possible hurdles in their implementation is conducted in parallel with the claims data analysis. Best practice models and, if applicable, existing empirical results (especially with regard to the acceptance of users) are to be analyzed. The systematic review is conducted in accordance with PRISMA (Preferred Reporting for Systematic Reviews and Meta-Analyses) guidelines [17]. Literature research is performed in PubMed and Embase. The search strategy is developed using the PICO scheme [18]. Relevant search, MeSH (Medical Subject Headings) or Emtree, terms are assigned to each category and linked with Boolean operators. The search is limited to publications from 2011 onward and to articles written in English and German. The study selection is divided into the steps of title, abstract, and full-text screening. To ensure an objective approach, literature screening, as well as extraction, is carried out by 2 researchers independently of each other. Studies are included according to the following criteria: the study deals with the implementation of video consultation in the form of video-based consultation via web in real time between the patient and the medical or psychotherapeutic service provider in outpatient care, and the study focuses on useful fields of application and promoting/inhibiting factors. Since a very high heterogeneity of the results is assumed, a qualitative information synthesis of the results of the included studies on possible implementation factors, encouraging features, and barriers is performed using the program MAXQDA (VERBI).

### Focus Groups (C)

At the end of the first phase, 5 focus group discussions with relevant stakeholders on potential applications of video consultation and barriers to its use are conducted. The interim results of the claims data analysis and the systematic literature review are reviewed in order to achieve a well-grounded basis for the development of the interview guidelines for the focus groups.

Due to the uncertainties associated with the coronavirus pandemic regarding the possibility of face-to-face meetings, all focus groups are conducted online. In 2015, Abrams et al [19] demonstrated that focus groups in an audio-visual format can achieve a similar richness of data as physical face-to-face focus groups, distinguishing them from purely written text-based forms.

Relevant stakeholders include representatives of insured individuals, physicians as well as psychotherapists, and the SHIF perspective. In order to adequately take into account the differences between urban and rural areas, for each region type 2 focus groups are conducted for both the perspective of insured individuals and that of the service providers in order to include content relevant to urban and rural regions.

The recruitment of physicians and psychotherapists is carried out by the participating ASHIP, and interested insured persons are recruited by a cooperating self-help association. Participants of the payer perspective are recruited by the participating SHIF themselves. The composition of the focus groups with insured individuals should take into account characteristics such as age group, gender, presence of a chronic disease, and video consultation users/nonusers. For the focus groups with physicians and psychotherapists, relevant characteristics are age group, gender, and specialist group. The focus groups are led by a team of moderators, as described by Krueger and Casey [20], and take approximately 60 minutes per stakeholder group. The basis for the focus groups are semistructured interview guides that are derived from the content-related preliminary work of the systematic literature research. The course of the focus groups is video-recorded and transcribed afterward. On this basis, a qualitative content analysis according to Mayring [21] is carried out using the software for qualitative data analysis MAXQDA (VERBI).

### Design of the Surveys Including Discrete Choice Experiments (D-F)

Preferences play an increasingly important role in health care decision-making [22]. However, the complexity of health-related decisions poses a challenge due to the multitude of alternatives available. In such circumstances, the DCE method has been widely used for preference elicitation in health care [23]. DCEs are based on Random Utility Theory, a theory of human preference behavior that assumes respondents behave in a manner that maximizes their utility. Therefore, econometric models based on Random Utility Theory can be used to analyze data from DCE surveys. It is assumed that services or goods can be valued on the basis of the characteristics (called attributes) that determine them [24]. DCEs usually consist of a number of choice sets that represent hypothetical options as alternatives. Each choice set is composed of a set of attributes and each attribute is described by values (called levels). By asking respondents to choose between the choice alternatives, preferences are determined. The respondents’ choices are then used to derive the importance of the attributes and levels in terms of overall utility [25]. DCEs support the design of health programs and the prediction of demand and acceptability [23].

Based on the preliminary work, namely literature searches and focus groups, the survey and the DCEs are constructed. The DCEs are designed to elicit preferences for the design of video consultations of insured persons as well as physicians and psychotherapists. Relevant attributes and their levels are therefore determined in the first study phase. Following, choice alternatives are modeled using SAS (version 9.4; SAS Institute Inc).

The choice set (which covers multiple choice alternatives) should take into account design recommendations by Huber and Zwerina [26]. Generally, choice sets should not contain any stimuli for which 1 option is clearly advantageous and therefore does not require weighing. According to The Professional Society for Health Economics and Outcomes Research—ISPOR—recommendation on conjoint analyses, the maximum number of pairwise comparisons (tasks) that each respondent should answer should be between 8 and 16 [27]. In order to limit the number of stimuli per respondent, it is possible to use optimal designs in which, based on certain quality criteria, an appropriate subset (fractional design) is selected from the set of theoretically possible stimuli (full-factorial design) and thus a minimum number of pairwise comparisons is required [28]. Additionally, if the number of calculated tasks exceeds ISPOR recommendations, the questionnaire could be split into 2 or more blocks as response efficiency could decrease otherwise [27].

The DCE is embedded in a survey which is structured into three sections: (1) possible barriers and promoting factors for the use of video consultations, (2) preference survey using DCE, and (3) sociodemographic information. Additionally, for the insured individuals, health-related information is requested and for the physicians or psychotherapists, information on the medical profession are asked in the third section.

Prior to the distribution of the questionnaires, they are subjected to a pretest and revised accordingly. The methods of think-aloud and probing are used to determine comprehensibility, manageability, completeness, and the time required for completion [29].

To determine the sample size, the rule of thumb according to Reed and Orme [30] is applied. Under the further assumptions of generating multiple questionnaire versions with different blocks, an estimated 10% response rate, and in order to enable differentiated statements about subgroups, around 33,000 insured persons have to be contacted. Applying the previously described rule of thumb, possible blocking, 10% response rate, and possible subgroup analysis for the survey of physicians and psychotherapists 31,900 individuals are contacted. The final sample size depends on the results of claims data analysis and systematic literature review in order to form relevant subgroups.

The surveys are carried out with randomly selected insured persons—under the application of defined inclusion and exclusion criteria—by the participating SHIF and with all physicians as well as psychotherapists of the ASHIP who are allowed to provide video consultations in principle.

Inclusion criteria for the survey of insured individuals contain (1) insurance by 1 of the 3 participating SHIF, (2) 18 years or older, and (3) place of residence in 1 of the 4 selected regions as done in claims data analysis (A). Insured persons under legal guardianship, with a high need for nursing care (“Pflegegrad 4” and higher), nursing home residents, patients in palliative care, patients diagnosed with dementia, and insured persons whose data may not be used for research are excluded.

The participating SHIF and ASHIP contact their insured and members by means of a short letter, which includes further information on the study, the survey itself for paper-participation as well as an invitation to participate web-based via QR code.

The survey starts in November 2022 and ends at the end of March 2023. If necessary, a reminder is used to increase the response rate.

The survey is analyzed in terms of possible barriers and preferences. Econometric analysis follows the ISPOR guidance for DCEs [31]. Descriptive procedures as well as logistic regression analyses are carried out. The target variable of the DCE is the subjects’ choice decision for 1 of 2 stimuli. Depending on the model, the attributes or their characteristics as well as sociodemographic and health-related characteristics of the test persons are included as explanatory variables. A mixed logit model is estimated. A hierarchical Bayesian model is used for the main analysis.

### Workshop With Stakeholders (G) and Health Policy Recommendations (H)

Finally, a stakeholder workshop (G) is performed to combine all findings of prior sections and summarize results. Participants include 1 representative each from the participating associations of SHI-accredited physicians, the participating SHIF and the participating cooperation partners of the self-help associations, patient representatives, the German Association of Medical Specialists, and the Professional Association of German Internists. Participants are asked to sign a declaration of consent. The workshop is held in person with an estimated duration of 6 hours and is led by a team of moderators from the University of Duisburg-Essen.

Finally, the results of the workshop are implemented in a blueprint with health policy recommendations (H).

### Ethical Considerations

The study received ethical approval from the Ethics Committee of the Medical Faculty of the University of Duisburg-Essen on September 27, 2022 (reference: 21-10283-BO). For the purpose of claims data analysis, the University of Duisburg-Essen only has access to aggregated data. There is no possibility of participant identification. The data collected in the focus groups is considered pseudonymized, with only statements needed in connection with the project being stored. The list of participants is deleted after the focus groups have been conducted and the audio recordings are deleted after transcription. For the participation in focus groups participants are compensated with 50 Euro (US $49.98) for insured individuals and 120 Euro (US $119.952) for physicians as well as psychotherapists. To take part in the surveys or the workshop, participants are asked to sign a declaration of consent.

## Results

As the study runs until March 2024, the methodological approach used in this study is the focus of this paper. The claims data analysis (A) demonstrates that video consultation has had almost no relevance in outpatient care in the German health care system from 2017 to 2019. This changes significantly with the beginning of the COVID-19 pandemic. Displaying the initial situation gives insights into areas of application and user groups which are published separately. Furthermore, the analysis is a necessary basis for further work.

The systematic research (B) on possible applications of video consultation and possible hurdles as well as promoting factors also aims to identify possible attributes and their levels for the DCE. Challenging aspects of the video consultation have been identified. In-depth descriptions of hurdles and promoting factors on the use of video consultation are published separately.

Focus groups (C) complement the systematic literature research to discuss the relevance of the attributes identified, as well as the levels and their scaling, which supported developing additions or modifications if necessary. They have been conducted with insured persons as well as with physicians and psychotherapists from rural and urban regions. This was supplemented by a focus group with representatives of SHIF.

The conception of the surveys (D) to collect preferences for the design of video consultations by insured individuals as well as by physicians and psychotherapists has been completed. Extensive pretests have been conducted. In addition, the questionnaires have been implemented on the web-based survey platform Question Pro for the additional option of digital participation, so that the recruitment of survey participants could begin in the fourth quarter of 2022 and was completed in March 2023 (E). The data are analyzed since April 2023 (E).

## Discussion

Video consultation is a promising tool in medical and psychotherapeutic care, as shown by its increasing use with the onset of the COVID-19 pandemic in claims data analysis (A) and more generally in light of advancing digitalization [32]. The study is directly designed for practical use to further develop the potential of video consultation in Germany. To the authors’ knowledge, a comprehensive approach to analyzing the use and provision of video consultations from the providers’ perspective on the one hand and the perspective of insured individuals on the other hand in 1 study has not been conducted to date in Germany. The results of this study will specifically address the conditions (expectations of form and content, inhibiting and promoting factors) under which the insured individual accepts the digitalization of their medical consultations and which application possibilities of video consultation are accepted by medical and psychotherapeutic service providers (differentiated by specialist groups). This is accomplished by taking into account possible barriers and considering the differences between rural and urban regions in order to be able to apply the defined recommendations in a targeted manner. Recommendations for legal and sublegal adjustments are then derived from this.

However, as this study addresses the German SHI, the legal framework might differ elsewhere; thus, our results may not be completely applicable to other countries. The subject of this study is video consultation and does not include telephone care or other types of telemedicine, which limits the perspective of this research.

Regardless of the topic addressed by this study (video consultations in outpatient medical care in Germany), the iterative and mixed methods approach used in this study protocol is also suitable for a variety of other research projects. The use of a systematic literature review as a first step to examine settings for video consultation and possible barriers ensures a systematic inclusion of the current state of the art while also taking into account the quality of the literature. The explorative elicitation of initial information on the basis of qualitative methods (in this case focus groups, which can also be supplemented or replaced by expert interviews) in the first study phase as a solid basis for the second study phase, in which the exploratively collected theses are to be examined quantitatively, is suitable for a wide range of inquiries in health services research. Furthermore, as urban and rural settings are explored, the study in general contributes to a better understanding of urban and rural differences in preferences in the provision of digital care. This can help policy makers in finding suitable solutions for both the rural and the urban population.

## Abbreviations

ASHIP: associations of statutory health insurance physicians
DCE: discrete choice experiment
GP: general practitioner
MeSH: Medical Subject Headings
PRISMA: Preferred Reporting for Systematic Reviews and Meta-Analyses
SHI: statutory health insurance
SHIF: statutory health insurance fund

## Data Availability

The data sets generated or analyzed during this study are not publicly available due to data protection reasons.
